# *CYP1A2* polymorphism −1545C > T (rs2470890) is associated with increased side effects to clozapine

**DOI:** 10.1186/1471-244X-14-50

**Published:** 2014-02-20

**Authors:** Merja Viikki, Olli Kampman, Niko Seppälä, Nina Mononen, Terho Lehtimäki, Esa Leinonen

**Affiliations:** 1 University of Tampere, Medical School, University of Tampere, Tampere 33014, Finland; 2 Tampere Mental Health Centre, Hallituskatu 8 B, 33200 Tampere, Finland; 3 Seinäjoki Hospital District, Department of Psychiatry, Seinäjoki, Finland; 4 Department of Psychiatry, Satakunta Hospital District, Fi-28500, Pori, Finland; 5 Department of Clinical Chemistry, Fimlab Laboratories, School of Medicine University of Tampere, Tampere, Finland; 6 Department of Psychiatry, Tampere University Hospital, Tampere, Finland

**Keywords:** 1545C > T, rs2470890, Clozapine, Side effects, Antipsychotic

## Abstract

**Background:**

Cytochrome P450 1A2 gene (*CYP1A2*) polymorphisms have been suggested to be associated with increased side effects to antipsychotics. However, studies on this are scarce and have been conducted with either various antipsychotics or only in small samples of patients receiving clozapine. The aim of the present study was to test for an association between the *CYP1A2 −*1545C > T (rs2470890) polymorphism and side effects in a larger sample of patients during long-term clozapine treatment.

**Methods:**

A total of 237 patients receiving clozapine treatment completed the Liverpool University Neuroleptic Side-Effect Rating Scale (LUNSERS) assessing clozapine-induced side effects. Of these patients, 180 completed the questionnaire satisfactorily, agreed to provide a blood sample, and were successfully genotyped for the polymorphism.

**Results:**

The TT genotype of *CYP1A2* polymorphism −1545C > T (rs2470890) was associated with significantly more severe side effects during clozapine treatment (*p* = 0.011). In a subanalysis, all seven types of side effects (sympathicotonia–tension; depression–anxiety; sedation; orthostatic hypotension; dermal side effects; urinary side effects; and sexual side effects) appeared numerically (but insignificantly) more severely among TT carriers. In addition, use of mood stabilizers was more common among patients with the TT genotype (OR = 2.63, *p* = 0.004).

**Conclusions:**

This study has identified an association between the *CYP1A2* polymorphism −1545C > T (rs2470890) and the occurrence of more severe clozapine side effects. However, these results should be regarded as tentative and more studies of larger sample sizes will be required to confirm the result.

## Background

Clozapine is the most effective antipsychotic in treatment refractory schizophrenia [1]. However, its use has been limited because of the documented infrequent, but serious side effects such as agranulocytosis. Several other less serious but more frequent side effects are also noted including sedation, weight gain, constipation, hypersalivation, and hypotension, which may lead to an impaired quality of life or the need to discontinue clozapine treatment.

Clozapine is metabolized mainly by the cytochrome P450 enzyme CYP1A2 [2]. The wide inter-individual variation in the expression and activity of CYP1A2 suggests a role for genetic factors in its control such as single nucleotide polymorphisms, as well as epigenetic and environmental factors, including smoking, coffee drinking, and co-medication [2-4]. The genetic polymorphisms of *CYP1A2* may affect clozapine clearance and plasma levels. For instance, the *1 F allele is associated with increased enzyme inducibility and the *1C allele with decreased inducibility [5,6]. In addition, poor clozapine response and low plasma drug levels have been found in smokers with the −163A/A genotype [7,8].

*CYP1A2* polymorphisms have previously been suggested to be associated with the side effect response to antipsychotics. An increased tardive dyskinesia severity was observed in patients with the A allele of the CYP1A2*1C polymorphism and in another study with CYP1A2 (C→A) [9,10], while the *CYP1A2* −163C > A polymorphism is associated with clozapine-induced generalized tonic-clonic seizures [11]. It is postulated that the concomitant absence of CYP1A2*F and presence of CYP1A2*C could generate *CYP1A2* phenotypes that induce lower mRNA expression and make patients more susceptible to clozapine intolerance [12]. It is possible that the *CYP1A2* polymorphism −1545C > T could play some role in it. Recently, Ferrari et al. [13] reported that patients with adverse drug reactions to clozapine had a higher frequency of the *CYP1A2* low-activity allele and lower *CYP1A2* mRNA levels than patients without adverse effects [13]. To the best of our knowledge, however, only one previous study has reported a possible association between the −1545C > T (rs2470890) polymorphism and the side effects of antipsychotics; in this analysis, 1545C > T was initially associated with tardive dyskinesia, but, after multiple corrections, this finding was shown to be insignificant [14].

Interesting findings on the association between *CYP1A2* and the side effects of clozapine are scarce, and such studies have been conducted on fairly small samples. The aim of the present study, therefore, was to test for an association between the *CYP1A2* polymorphism −1545C > T (rs2470890) and side effects in a larger sample of patients during long-term clozapine treatment.

## Methods

### Patients

The study included 237 patients with diagnoses of schizophrenia, schizophreniform, schizoaffective, or delusional disorders according to the International Classification of Diseases, Tenth Revision (ICD-10). The diagnoses were set by experienced psychiatrists. All patients were ≥18 years of age, Caucasian, of Finnish origin, and were receiving clozapine treatment. Patients who had used clozapine for less than three months or whose mental state did not allow reliable self-assessment were excluded from the study. Of the 237 patients, four were excluded because of incomplete LUNSERS data (at least 20% of responses missing). A total of 190 patients (112 men and 78 women) provided samples for the commercial laboratory measurement of serum clozapine and norclozapine concentrations, and 187 patients were successfully genotyped for −1545C > T (rs2470890).

The study was conducted in three hospital districts in Western Finland (Satakunta, Pirkanmaa, and Seinäjoki). The patients were recruited at secondary in- and outpatient clinics and from sheltered accommodation units. All participants (*n* = 237) were asked to complete the Liverpool University Neuroleptic Side-Effect Rating Scale (LUNSERS) [15]. This is a self-reported questionnaire of 51 items that assess the intensity of antipsychotic side effects (0 = not at all, 4 = very much). Forty-one of the items assess antipsychotic-induced side effects while the remaining 10 serve as “red herrings” to detect patients who may be over-reporting symptoms. The reliability and validity of LUNSERS is well-established [16], although its validity has not been confirmed in relation to the use of atypical antipsychotics. Therefore, we performed an analysis to detect clinically meaningful factors for different types of side effects. This identified eight clinical factors: sympathicotonia–tension, depression–anxiety, sedation, orthostatic hypotension, dermal side effects (rash, sensitivity to sun, new or unusual skin marks or itchy skin), urinary side effects (difficulty passing urine or passing a lot of urine), sexual side effects (increased or decreased sexual drive or difficulty in achieving orgasm), and menstrual side effects; the latter was omitted from this study. Information on medical history and duration of clozapine treatment were collected from medical records. The study was approved by the local ethics committee. All participants gave informed consent upon entry to the study. This study was carried out in accordance with the code of Ethics of the World Medical Association (Declaration of Helsinki).

### DNA extraction and genotyping

For DNA extraction, 9 ml EDTA-whole blood was drawn from the participants and stored in a freezer at −20°C. Genomic DNA was extracted from peripheral blood leukocytes using the QIAamp^®^DNA Blood Minikit and automated biorobot M48 extraction (Qiagen, Hilden, Germany). Genotyping of rs2470890 was performed using the Taqman^®^SNP Genotyping Assay (assay C_1642455_10) and ABI Prism 7900HT Sequence Detection System (Applied Biosystems, Foster City, CA, USA). Pipetting for genotyping was performed on 384 plates by a Tecan Evo Freedom robot with eight pipetting tips.

### Clozapine assay methodology and blood sampling

Clozapine serum concentration measurements were taken during concomitant routine leukocyte monitoring. The patients were instructed not to eat or take medications before the laboratory test, which took place in the morning. This would then achieve a medication-free period of 8–14 h prior to serum clozapine concentration measurement. Clozapine serum levels were determined by high performance liquid chromatography with ultraviolet detection (described in detail in [17]).

### Statistical analyses

Analysis of variance and *t*-tests were used for LUNSERS total scores, factor scores, clozapine dose, and concentration comparisons between different *CYP1A2* genotype groups. Chi-square statistics were used to calculate differences between frequency of medication use and *CYP1A2* genotypes. The general linear univariate model (analysis of covariance) was used to determine the effects of antipsychotic doses and *CYP1A2* genotype on LUNSERS total score. In the model, the antipsychotic dose was used as a covariate and the *CYP1A2* genotype as a factor. In alternative models, the following factors were also used one at a time: clozapine monotherapy, antipsychotic combination, or regular smoking. Power calculations were made between the LUNSERS total score and *CYP1A2* genotypes according to realized distributions in these variables. This resulted in a power of 0.803 with a probability (*p*) < 0.05. All calculations were performed with SPSS (version 19) and PS (version 3.0.43; [18]) software.

## Results

A total of 187 patients (110 men [61%] and 70 women [39%]) were successfully genotyped for the −1545C > T (rs2470890) polymorphism; of these, 185 had a valid LUNSERS rating. The mean age was 43.1 (±11.0) years (range, 20–67 years).

The majority of patients had been receiving clozapine for over five years (64%). Out of all patients, 34% had used clozapine for 1–5 years and only 1.9% had used it for less than a year (3–12 months). The mean time from the first episode of hospitalization for psychosis until the start of the study was 17.3 (±10.0) years.

The TT genotype of *CYP1A2* polymorphism −1545C > T (rs2470890) was associated with a significantly greater side effect frequency during clozapine treatment as mean LUNSERS scores were 45.1, 36.8, and 37.9 in TT, TC, and CC carriers, respectively (*p* = 0.038), and 45.1 vs. 37.1 among TT carriers vs. TC or CC carriers (*p* = 0.011). In a subanalysis, all seven types of side effects were found to be more severe among TT carriers, but this finding was not statistically significant (Table 1).

**Table 1:** LUNSERS scores (mean ± SD) and frequency of side effects according to genotype and in the total population

**Symptom**	**LUNSERS scores mean (±SD)**			
	**Frequency of the symptom %**			
	**TT (n=54)**	**TC (n=96)**	**CC (n=35)**	**Total (n=185)**
Sympathicotonia-tension	7.35 (±4.87)	6.54 (±4.77)	6.69 (±5.61)	6.81 (±4.95)
	88.9%	92.7%	82.9%	89.7%
Depression-anxiety	9.65 (± 5.94)	8.11 (±4.55)	8.14 (±5.14)	8.57 (±5.12)
	90.7%	91.7%	88.6	90.8%
Sedation	5.13 (± 3.28)	4.81 (±2.65)	4.97 (±2.83)	4.93 (±2.87)
	88.9%	96.9%	91.2%	93.5%
Orthostatic hypotension	3.41 (±2.67)	2.79 (±2.51)	2.82 (±2.86)	2.98 (±2.63)
	81.5%	75.0%	70.6%	76.1%
Dermal side effects	1.11 (±1.46)	1.09 (±1.62)	0.97 (±1.43)	1.08 (±1.53)
	47.3%	40.6%	38.2	42.2%
Sexual side effects	1.96 (±2.23)	1.61 (±1.99)	1.73 (±1.94)	1.74 (±2.05)
	61.8%	54.2%	59.5%	57.4%
Urinary side effects	2.09 (±1.94)	1.45 (±1.90)	1.80 (±2.25)	1.70 (±1.99)
	74.5%	49.0%	54.1%	57.4%

The use of mood stabilizers was significantly more common among patients with the TT genotype than with TC/CC (OR = 2.63, *p* = 0.004) (Table 2). Of the patients, 65% (*n* = 121) were receiving clozapine monotherapy, 24% (*n* = 45) received a combination of clozapine and second generation antipsychotic therapy (most often aripiprazole), 9.6% (*n* = 18) received a combination of clozapine and first generation antipsychotic therapy, and 1.6% (*n* = 3) received both first and second generation antipsychotic therapy in addition to clozapine. The clozapine or norclozapine concentration (or their sum or ratio) did not differ between TT carriers and CC/TC carriers. In general linear univariate analysis, the interaction between total antipsychotic dose and the genotype of *CYP1A2* −1545C > T (rs2470890) together explained 5.0% of the variance in the LUNSERS scale total score (complete model: *p* = 0.037, power = 0.682; antipsychotic dose: ηp2 = 0.018, *p* = 0.08; *CYP1A2* genotype: ηp2 = 0.035, *p* = 0.051). The results of alternative models, in which clozapine monotherapy, antipsychotic combination, or regular smoking were taken as factors one at a time were non-significant and the additional factors had a low predictive power in these models.

**Table 2:** **Comparisons between *CYP1A2 *** **1545C > T (rs2470890) TT and C-carrier patient groups**

**Patients**	**TT (n=54)**	**TC/CC (n=133)**	**PP between groups**
Males	32 (59.3%)	78 (58.6%)	NS
Regular smokers	29 (52.7%)	65 (49.6%)	NS
Antipsychotic co-medication	18 (33.3%)	48 (36.1%)	NS
Atypical	14 (25.9%)	31 (23.3%)	
Conventional	2 (3.7%)	16 (12.0%)	
Both	2 (3.7%)	1 (0.8%)	
Mood stabilizer adjuvant therapy	24 (44.4%)	31 (23.3%)	0.004
SSRI/SNRI adjuvant therapy	21 (38.9%)	46 (34.6%)	NS
Clozapine + norclozapine serum level mol/l (mean+SD)	2.21+0.98	2.45+1.36	0.20
Clozapine/norclozapine ratio (mean+SD)	1.68+0.60	1.71+0.59	0.78
Clozapine dose/serum level ratio (mean+SD)	441+195	405+309	0.44
Clozapine dose mg (mean+SD)	431+132	390+150	0.078
Antipsychotic total dose mg* (mean+SD)	971+380	883+389	0.17

*In chlorpromazine equivalents.

The allele frequencies in the patient sample were equal to those in the general population (T = 0.54, C = 0.46), and there was no gender difference in allele distributions (men T = 0.56, women T = 0.53; *p* = 0.55, chi-square test). The genotypes in the sample were in Hardy-Weinberg equilibrium (*p* > 0.10).

## Discussion

In the present study, the TT genotype of *CYP1A2* polymorphism −1545C > T (rs2470890) was associated with an increased frequency of side effects during clozapine treatment. In addition, a subanalysis of results showed that all seven types of side effects (sympathicotonia–tension; depression–anxiety; sedation; orthostatic hypotension; dermal side effects; urinary side effects; and sexual side effects) were more severe among TT carriers, but this finding was not statistically significant. Previous studies reported that different *CYP1A2* polymorphisms were associated with adverse effects of antipsychotics; although, to our knowledge, only one has assessed the association between the −1545C > T (rs2470890) polymorphism and antipsychotic side effects [14]. Moreover, in this study, an excess of the T allele was observed among patients with tardive dyskinesia during initial analysis, but this was later revised after multiple corrections to be an insignificant finding [14]. Our results are in line with the initial analysis of this earlier work.

In previous studies, higher plasma concentrations of clozapine and its metabolite *N*-desmethylclozapine have been reported in patients with two *CYP1A2* variants associated with reduced enzyme activity (−3860A, −2467del, −163C, −739G, and/or −729T) compared with those with one or no variants [19]. Although it might be expected that the higher frequency of side effects among TT carriers observed in the present study could be explained by higher plasma concentrations of clozapine in those individuals, we found no difference in clozapine or norclozapine concentrations (or their sums or ratios) between TT carriers and CC/TC carriers. Likewise, use of other co-medication, such as selective serotonin uptake inhibitors (SSRIs) and serotonin norepinephrine reuptake inhibitors (SNRIs) and other antipsychotics may have contributed to the frequency or severity of side effects. However, the use of SSRIs, SNRIs, and the total dose of all antipsychotics did not differ between patients with the TT genotype and those with CC/TC genotypes.

Smoking is a known *CYP1A2* inducer, and lower plasma concentrations of clozapine have previously been detected in smokers with the −163A/A genotype [7,8,20]). Since the C allele of CYP1A2*1 F, which is in complete linkage disequilibrium with the C allele of CYP1A2 1545C > T (exon) [14], leads *to* decreased inducibility among smokers, the C allele of rs2470890 would also be expected to be associated with decreased inducibility. Moreover, if smoking was more common among patients with the TT genotype in the present study, this would explain why such individuals require higher clozapine doses yet serum clozapine concentrations are not increased. However, smoking status was similar in all patients. Another lifestyle factor that could explain the difference in CYP1A2 metabolism is coffee drinking. It has been suggested that caffeine inhibits clozapine metabolism and increases serum clozapine concentrations [21]. Although caffeine intake was not assessed in the present study, the effect of drinking instant coffee on serum clozapine concentrations was reported to be of minor clinical relevance in most cases within a small sample of Finnish patients [22]. Carbamazepine, used to control epileptic seizure, also induces *CYP1A2*[23]. However, it was seldom used in our patients and there was no difference in the frequency of carbamazepine use between TT carriers and CC/TC carriers.

The second finding of the present study was that mood stabilizer use was more common among individuals with the TT genotype than CT or CC carriers. The reason for this is unclear, although it is possible that the TT carriers who experienced more adverse effects also complained more about other symptoms such as anxiety, depression, or mood swings. This may have resulted in the administration of a mood stabilizer, and this combination could have increased the frequency or severity of adverse effects. It is also conceivable that the TT genotype is associated with *CYP1A2* induction, thus weakening the antipsychotic response and requiring the addition of a mood stabilizer. However, the most common reasons for adding a mood stabilizer were to prevent seizures, as clozapine increases their risk, and to reduce aggression. In some cases, mood stabilizer use was indicated because the primary disease was schizoaffective disorder. However, only 6% (*n* = 14) of patients had a diagnosis of schizophreniform, schizoaffective, or delusional disorders. It was not possible to analyze these disorders separately, however, and this can be regarded as a study limitation. The most commonly added mood stabilizer in the present study was valproic acid.

The assay for clozapine measurement provides excellent precision and accuracy. However, it has been suggested that the thermal instability of clozapine-*N*-oxide represents a concern regarding the potential for overestimation of clozapine [24]. When exposed to heat or to alkaline conditions clozapine-*N*-oxide again yields clozapine. However, this was unlikely in the present study because special efforts were taken during sample workup and chromatographic analysis to avoid overestimation of clozapine.

The main limitation of our study was its fairly small sample size, which markedly reduces the statistical power to detect small or moderate effects. However, we recruited almost all patients within the geographical area who were receiving clozapine treatment and able to give their informed consent. A second limitation is that LUNSERS is a self-reported questionnaire that requires items to be rated from “not at all” to “very much” with no description or criteria. This could have affected reliability. Moreover, the reported side effects were not systematically confirmed by clinicians or compared with clinical records, and we lacked data on which side effects led to reduction of the dose. Nevertheless, the reliability and validity of LUNSERS is well-established [16]. Most patients had been on clozapine for over 5 years, so there were no cases of agranulocytosis/neutropenia, which usually develops during the first 3 months of clozapine use; we also only included patients who had been on clozapine for at least 3 months. Weight gain was a common side effect, with 56% of patients reporting this although we did not analyze it here as this will be presented in a separate analysis. Lack of data on caffeine intake is also a limitation of the present study. The results of alternative statistical models, with clozapine monotherapy or antipsychotic combinations as individual factors, were non-significant and the additional factors had a low predictive power in these models. Finally, the lack of patient mean positive and negative syndrome scale data meant that we could not assess whether −1545C > T was related to lack of response.

## Conclusions

The present findings suggest an association between the TT genotype of the *CYP1A2* polymorphism −1545C > T (rs2470890) and side effects of clozapine. However, these findings should be regarded as tentative and more studies with larger samples will be required to confirm the results.

## Competing interests

The authors declare that they have no competing interests.

## Authors’ contributions

Authors EL, OK, NS and TL designed the study. Authors NS and MV participated in recruiting the patients. Authors MV, EL, OK, NS, NM and TL wrote the article. Author OK undertook the statistical analysis. All authors contributed to and have approved the final manuscript.

## Pre-publication history

The pre-publication history for this paper can be accessed here:

http://www.biomedcentral.com/1471-244X/14/50/prepub
